# Foreign body in nasopharynx: an accidental radiological finding

**DOI:** 10.1016/S1808-8694(15)30984-8

**Published:** 2015-10-19

**Authors:** Regina Helena Garcia Martins, Juliano B. Mano, Eriverton F. da Silva

**Affiliations:** a Assistant professor, Doctor in Surgery by the Botucatu Medical College - Unesp. Responsible for the phoniatrics and voice outpatient unit. Professor of Otorhinolaryngology at the Paulista State University - Unesp, Botucatu campus; b Medical resident in Otorhinolaryngology at the Botucatu Medical College, Unesp; c Medical resident in Otorhinolaryngology at the Botucatu Medical College, Unesp. Oftalmologia, Otorhinolaryngology and Head & Neck Surgery Departments. Otorrinolaringologia Unit of the Botucatu Medical College (UNESP)

**Keywords:** children, foreign bodies, nasopharynx

## INTRODUCTION

Upper airways and the upper digestive tract may harbor foreign bodies such as sponges, grains, toy parts, stones, paper, insects, cotton, etc^1^, ^4^. These objects may go undetected for week or even years. Foreign bodies in the nasopharynx are rare, with few citations in literature; they deserve to be highlighted owing to diagnostic difficulties and delays.

## CASE REPORT

**Figure 1:**
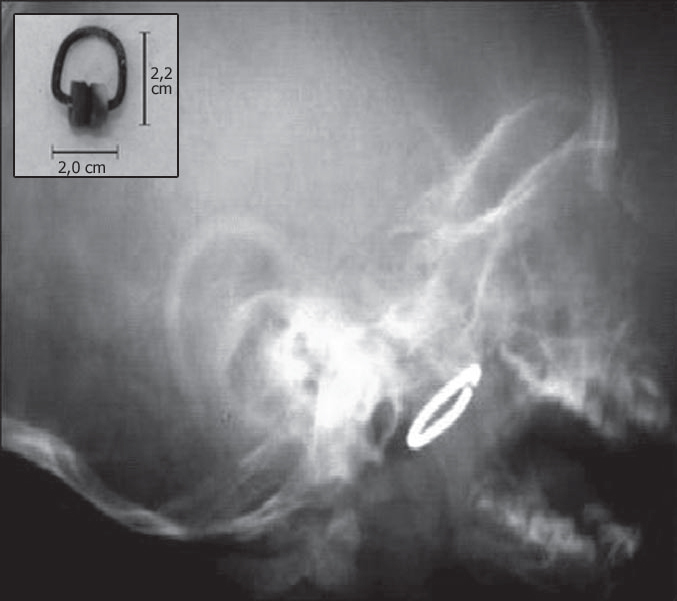
1a: radiologic exam showing a foreign body in the nasopharynx. 1b: curtain rail hook removed from the nasopharynx; observe the degree of oxidation of metal parts.

DRE, aged 1 year and 8 months, male, underwent a deglutogram to investigate gastroesophageal reflux, which identified a radiopaque object in the rhinopharynx (Figure 1a). The children lived in an orphanage and the caretaker could not inform details about symptoms, but reported bilateral otorrhea since the first few months of life and recurrent pneumonia (which had justified the investigation of reflux). The caretaker also reported that four months ago the child had a sudden episode of respiratory discomfort of no apparent cause, improving with “tapotement”. On physical examination, the child was afebrile, eupneic, with unobstructed nasal fossae, but with hialin secretion. Otoscopy confirmed bilateral tympanic perforation. The child was uncooperative, and was sedated to allow detailed examination of the cavum, which allowed us to digitally identify and remove a metal curtain rail hook measuring 2.0 × 2.2 cm, enveloped in thick secretion and with oxidized metal parts (Figure 1b).

## DISCUSSION

In the case we report, the probable route for the foreign body was accidental ingestion with progression to the glottic region (in an attempt to swallow the object, which could explain the dyspnea crisis) eventually lodging in the rhinopharynx thanks to the “tapotement” maneuvers by attendants. The foreign body had probably been in the rhinopharynx for some time due to the degree of oxidation. It did not seem the cause of otorrhea or recurrent pneumonia, as the children had these entities since early months of life.

Foreign bodies in the nasal fossae and rhinopharynx may cause purulent rhinorrhea, nasal obstruction, chronic rhinosinusitis, persistent coughing or may remain asymptomatic. Tay^5^ described a patient with an asymptomatic foreign body in the nasopharynx for 20 years. Frequently rhinopharyngeal foreign bodies are accidental findings on radiology, as with the present case. When inhaled, they may lodge in bronchi causing pneumonia, atelectasis and bronchiectasis, the main complication in late diagnosis^6^. The history is positive in approximately 70% of cases and of these, only 60% seek medical help within the first 24 hours^6^.

If a foreign body in the upper airways or the upper digestive tract is suspected, endoscopic and radiologic exams should be promptly done. The variety and sharpness of endoscopes have facilitated object recognition and removal, even when in locations that are difficult to access.

## FINAL COMMENTS

Foreign bodies in the nasopharynx are rare, usually seen in children, and may be asymptomatic. If suspected, endoscopic and radiologic investigation should be done, as these foreign bodies may be swallowed or aspirated, complications which are associated with high morbidity rates.

## References

[1] Ogut F, Bereketoglu M, Bilgen G, Totan S (2001). A metal ring that had been lodged in a child's nasopharynx for 4 years. Ear Nose Throat J.

[2] Saxena SK, Gopala Krishnan S, Rav D (2002). An unusual impacted foreign body in the nasopharynx. Indian J Otolaryngol Head Neck Surg.

[3] Eghtedari F (2003). Long lasting nasopharyngeal foreign body. Case Reports. Otolaryngol Head Neck Surg.

[4] Sobrinho FPG, Jardim AMB, Sant’Ana IC, Lessa HA (2004). Corpo estranho na nasofaringe: a propósito de um caso. Rev Bras Otorrinolaringol.

[5] Tay AB (2000). Long-standing intra nasal foreign body: an incidental finding on dental radiograph. Oral Surg Oral Med Pathol Oral Radiol Endod..

[6] Karakoc F, Karadag B, Akbenlioglu C, Eusu R, Yildzeli B, Yksee M, Dagli E (2002). Foreign body aspiration: what is the outcome?. Pediatr Pulmonol.

